# The Expression of Two Distinct Sets of Glycolytic Enzymes Reveals Differential Effects of Glycolytic Reprogramming on Pancreatic Ductal Tumorigenesis in Mice

**DOI:** 10.3390/biomedicines11112962

**Published:** 2023-11-02

**Authors:** Yannan Zhang, Fangfang Zheng, Fan Wang, Xingqian Liu, Cong Xiang, Shiyu Fu, Kun Shen, Geng Liu

**Affiliations:** State Key Laboratory of Pharmaceutical Biotechnology, MOE Key Laboratory of Model Animals for Disease Study, Jiangsu Key Laboratory of Molecular Medicine, Model Animal Research Center, School of Medicine, Nanjing University, 12 Xuefu Road, Pukou High-Tech District, Nanjing 210061, China; zhangyn@nicemice.cn (Y.Z.); zhengff@nicemice.cn (F.Z.); wangfan@nicemice.cn (F.W.); liuxq@nicemice.cn (X.L.); xiangcong@nicemice.cn (C.X.); fusy@nicemice.cn (S.F.); shenk@nicemice.cn (K.S.)

**Keywords:** pancreatic cancer, PDAC, PanINs, glycolysis

## Abstract

Pancreatic ductal adenocarcinoma (PDAC) is associated with enhanced aerobic glycolysis through elevated glucose uptake and the upregulated expression of genes encoding rate-limiting glycolytic enzymes. However, the direct impact of altered glycolytic pathways on pancreatic tumor progression has not been thoroughly investigated. Here, we utilized two strains of BAC transgenic mice with pancreatic expression of two distinct sets of glycolytic genes each arranged in a polycistronic fashion (*PFKFB3-HK2-GLUT1* and *LDHA-PDK1*, respectively) to investigate the role of altered glycolysis on the development of pancreatic ductal tumor development in the *Pdx1-Cre; LSL-Kras^G12D^* mice. The overexpression of the two sets of glycolytic genes exhibited no significant effects on tumor development in the 4–5-month-old mice (the PanIN2 lesions stage). In the 9–10-month-old mice, the overexpression of PFKFB3-HK2-GLUT1 significantly accelerated PanIN3 progression, exhibiting elevated levels of ductal cell marker CK19 and tumor fibrosis. Surprisingly, the overexpression of LDHA-PDK1 significantly attenuated the progression of PanIN3 in the 9–10-month-old mice with significantly downregulated levels of CK19 and fibrosis. Therefore, distinct set of glycolytic enzymes that are involved in different glycolytic routes exhibited contrasting effects on pancreatic ductal tumor development depending on the tumor stages, providing novel insights into the complexity of the glycolytic pathway in the perspective of PDAC development and therapy.

## 1. Introduction

Pancreatic cancer (PC) is the fourth most common cause of cancer-related deaths worldwide with a poor prognosis, particularly with a median survival of 3–6 months and a 5-year survival rate of less than 4% [1,2]. Despite only representing <3% of all cancer diagnoses, it is predicted to become the second leading cause of cancer deaths by 2030 [3]. Pancreatic ductal adenocarcinoma (PDAC), the most common subtype of PC, is an invasive mucinous adenoma. The majority of PDAC cases arise from non-invasive pancreatic ductal epithelial cell proliferation, which is called pancreatic intraepithelial neoplasias (PanINs) [4].

The driving oncogene in PanINs and PDAC is an activating mutation in the Kras gene found in >90% of tumors [5,6]. The mutated Kras activates the downstream signaling pathways sustaining cell proliferation, metabolic reprogramming, and tumor microenvironment rearrangement. Being the most commonly used mouse model of pancreatic cancer, the *Pdx1-Cre; LSL-Kras^G12D^* mice spontaneously develop pancreatic ductal intraepithelial neoplasia, highly mimicking the progression of human pancreatic tumors [7]. In this mouse model, PanINs lesions can be divided into three levels according to the differences in cell morphology, including PanIN1A/1B, PanIN2, and PanIN3 lesions [4,8]. PanIN1A/1B and PanIN2 lesions occurred at 2.25 and 4.5 months of age in mice, respectively, and 9-month-old mice showed a high degree of malignant PanIN3, but only a small number of mice developed PDAC [9,10]. In addition, PDAC is linked to significant fibrosis [11]. The major cell types responsible for fibrosis are pancreatic stellate cells (PSCs), which activate alpha-smooth muscle actin (α-SMA) expression and produce the collagenous stroma that promote tumor cell proliferation and migration [12,13]. Upon the emergence of early PanIN lesions, α-SMA and collagen levels were significantly elevated and associated with the emergence of pancreatic epithelial abnormalities [14].

Alterations in cell metabolism are one of the hallmarks of cancers that sustain cell growth and proliferation. Cancer cells metabolize glucose by aerobic glycolysis rather than through the more energetically efficient oxidative phosphorylation, even in the presence of oxygen, known as the Warburg effect [15]. Although the efficiency of ATP production is relatively low, the Warburg effect can convert glycolytic intermediates into various biosynthetic pathways, such as amino acids, lipids, and proteins [16]. PDAC is a highly glycolytic tumor, and a series of critical metabolic enzymes involved in glycolysis are abnormally overexpressed in PDAC [6,17,18]. The concurrently elevated expression of the glycolytic enzymes suggests that they might promote tumor glycolysis in a coordinated fashion. How the glycolytic enzymes that are possibly involved in different glycolytic routes impact metabolic reprogramming and tumor development remain not completely understood.

Glucose transporter 1 (GLUT1) is the most common glucose transporter in humans, and Hexokinase 2 (HK2) catalyzes the first step of glucose metabolism [19,20]. PFKFB3 synthesizes fructose 2,6-bisphosphate (F2,6BP) to activate 6-phosphofructo-1-kinase (PFK1), which mediates a key step in glycolysis [21]. These enzymes promote the rate of glycolysis through the augmented capacity for glucose uptake and fueling downstream pathways [19,20,21,22]. We have previously shown that the overexpression of the *PFKFB3-HK2-GLUT1* polycistronic gene set in mouse skeletal muscles greatly enhanced the glycolytic capacity and improved systemic glucose disposal [23]. Lactate dehydrogenase (LDHA) regulates the conversion of pyruvate to lactate as part of the glycolytic pathway [24]. Pyruvate dehydrogenate kinase 1 (PDK1) phosphorylates and inhibits components of the pyruvate dehydrogenase (PDH) complex that converts pyruvate produced from glycolytic flux to acetyl-CoA in order to suppress the flow of glycolysis to the mitochondrial TCA cycle [25,26]. Thus, the expression of LDHA-PDK1 in tandem may promote glycolysis by switching the glucose metabolic routes away from mitochondria.

Alterations of metabolic genes and metabolites offer not only new biomarkers for diagnosis and prognosis but also potential new targets for cancer therapy. In this study, we performed the pancreatic overexpression of two distinct sets of glycolytic genes *PFKFB3-HK2-GLUT1* and *LDHA-PDK1*(each arranged in tandem), respectively, in a *Pdx1-Cre; LSL-Kras^G12D^* mouse model to examine their impacts on the development of pancreatic tumors.

## 2. Materials and Methods

### 2.1. Mouse Generation and Animal Care

The construction of BAC vectors and generation of the BAC transgenic mice (BAC-Tg *LDHA-PDK1*) were performed as previously described [23]. Briefly, the human *LDHA* (NM001135239) and *PDK1* (NM001278549) full-length ORFs were obtained via PCR using human cDNA as templates. Myc tag and Flag tag were added to the 5′ end of LDHA and PDK1 ORFs, respectively. The two tagged genes and EGFP cassette were ligated with a different virus 2A sequence [27]. To control the expression of transgenes in a temporal- and spatial-specific manner, we constructed the expression plasmid containing the polycistronic cassette combined with the Cre-LoxP recombination system and the Tet-On inducible expression system (pRosa-CAG-rtTA-TRE-LSL-Myc-LDHA-E2A-Flag-PDK1-T2A-EGFP-WPRE). The linearized pRosa-CAG-rtTA-TRE-LSL-Transgenes vector was inserted into the ROSA26 BAC through homologous recombination. The ROSA26-Transgene BAC was microinjected into the male pronucleus of the zygotes. BAC transgenic mice derived from the Model Animal Research Center of Nanjing University with a C57BL/6J background. The transgenic mouse line (BAC-Tg *PFKFB3-HK2-GLUT1*) was described previously [23]. Tg mice were crossed with EIIA-Cre mice to generate STOP-deleted (referred to as Sd-Tg) transgenic mice. Pancreatic cancer model mice (*Pdx1-Cre; LSL-Kras^G12D^*, PK) were obtained by crossing *LSL-Kras^G12D^* mice and *Pdx1-Cre* mice, which were purchased from Jackson Laboratory. The BAC transgenic lines Tg *PFKFB3-HK2-GLUT1* and Tg *LDHA-PDK1* were crossed with PK mice to obtain PK; Tg *PFKFB3-HK2-GLUT1* mice and PK; Tg *LDHA-PDK1* mice, respectively. Genotyping was carried out using the primers listed in Table S1.

All mice were bred under specific pathogen-free conditions in a controlled environment of 20–22 °C, with a 12/12 h light/dark cycle and free access to food and water. Treatment with 0.2% doxycycline in drinking water was initiated in mice at 4 to 6 weeks of age. This study was authorized by the Institutional Animal Care and Use Committee (IACUC) of Model Animal Research Center, Nanjing University, China, and conducted in accordance with the guidelines of the IACUC and the approved Animal protocol #LG19.

### 2.2. Southern Blot

Southern blot was used to obtain the plasmids copy numbers of transgenic mice and to determine whether the plasmids were successfully integrated on the chromosomes. Genomic DNA was digested with MscI on a 0.8% agarose gel and transferred to a nylon membrane and hybridized with a cDNA probe for signal detection according to the standard method [28].

### 2.3. Cell Culture and Treatment

HEK-293T cells were transfected with the PFKFB3-HK2-GLUT1 and LDHA-PDK1 expression vectors, respectively, using Lipofectamine 2000 (Invitrogen, Carlsbad, CA, USA) according to the manufacturer’s instructions.

MEFs were isolated from E12.5 to E14.5 embryos from the transgenic mice as described previously [29]. Briefly, uteruses were carefully collected from pregnant female mice and placed in sterile PBS. The embryos were separated by slicing through the uterus walls between each embryo. The head and viscera of the embryos were removed and used for DNA extraction and genotyping PCR. The remaining parts of the embryo were minced and transferred to a 15 mL tube containing a mixture of 1 mL 0.25% trypsin and 1 mL PBS. The tube was incubated in a 37 °C water bath for 10 min with shaking every 3 min. After incubation, 8 mL of DMEM with 10% fetal bovine serum was added to the tube. The fetal tissues were vigorously pipetted until they were in a single-cell suspension. The cell suspension was then transferred to a fresh 10 cm culture dish and incubated in a 37 °C incubator with 5% CO_2_. The medium was changed after 12 h. These cells were considered to be passage 0. Because the early passage MEFs grow rapidly, they should be passaged and frozen when they reach 70–80% confluency.

HEK-293T cells and MEFs were cultured with DMEM (Gibco, Grand Island, NY, USA) supplemented with 10% FBS (Gibco, Thornton, NSW, Australia), 50 units/mL penicillin, and 50 µg/mL streptomycin (HyClone, Pasching, Austria) under 5% CO_2_ at 37 °C.

### 2.4. Immunohistochemistry (IHC)

Tissues were dissected and fixed overnight in ice-cold 4% paraformaldehyde and then transferred to gradient alcohol (from 70% to 100%), n-butanol, and embedded in paraffin. Sections were cut to a thickness of 6 µm. After deparaffinization and rehydration, sections were processed to heat-induced epitope retrieval with sodium citrate (pH 6.0) and were then blocked with normal goat serum at room temperature for half an hour. Next, sections were incubated with primary antibody followed by secondary antibodies labeled with avidin-tagged horseradish peroxidase (HRP). Signals were developed with DAB agents (MaiXinBio, Fuzhou, China), and then the nuclei were stained with hematoxylin. The antibodies used for immunohistochemistry were listed in Table S2.

### 2.5. Assessment of Primary Disease Burden

Mice were sacrificed, and the pancreas and other organs such as the livers and lungs were collected. The pancreas tissues were weighed and the tumor volumes were measured, immediately fixed in 4% buffered formalin, and then embedded in paraffin. For histopathological analysis, tissues were serially sliced (6 µm), and hematoxylin and eosin (H&E) staining was performed. The pancreatic intraepithelial neoplasia (PanIN) lesions were based on the criteria described previously [4,10]. To quantify PanIN lesions, approximately ten 100× magnification (objective lens 10×; eyepiece lens 10×) photographs were captured for the section with the largest cross-section of each tissue using the Olympus microscope (BX53), ensuring complete coverage of all the tissue areas. The Image J 1.52a software was used to statistically analyze the ratio of the PanIN lesions area to the total tissue area in each photograph. Subsequently, the average PanIN lesions area ratio in these photographs was determined as the PanIN lesions area ratio of the mouse pancreas.

### 2.6. Masson Trichrome Staining

Tissues from the mouse pancreas were fixed in 4% paraformaldehyde and cut into 6 µm thick sections. The sections were stained separately with Masson’s trichrome by using the Masson Trichrome Stain Kit (Servicebio, Wuhan, China) according to the manufacturer’s instructions. They were deparaffinized and rehydrated using xylene and gradient alcohol (from 100% to 70%) sequentially. Then, they were stained with iron hematoxylin solution, ponceau acid magenta, phosphomolybdic acid solution, and aniline blue dye for appropriate time sequentially. They were washed with distilled water after each staining. Finally, they were differentiated with 1% acetic acid solution for 2 min and dehydrated with anhydrous ethanol. The collagen fibers appeared blue, while the nuclei showed a bluish-black color. The red background revealed the presence of cytoplasm, muscle fibers, and cellulose. Pancreatic morphology and collagen deposition were observed under the microscope.

### 2.7. Western Blot (WB)

Cells and tissues were harvested, homogenized, and ground in ice-cold RIPA buffer containing Protease Inhibitor Cocktail Tablets (Roche, Mannheim, Germany). The concentration of the supernatant protein was also determined using the PierceTM BCA protein assay kit (Thermo, Rockford, IL, USA). Proteins were separated by electrophoresis and immunoblotted onto polyvinylidene fluoride membranes. Membranes were incubated with indicated primary antibodies overnight at 4 °C, followed by incubation with horseradish peroxidase-conjugated secondary antibodies for 2 h at room temperature. Detection was accomplished with High-sig ECL (Tanon, Shanghai, China), and signals were visualized using a Tanon 5200 automatic chemiluminescence imaging analysis system (Tanon, Shanghai, China). The antibodies for Western blot are listed in Table S3.

### 2.8. RNA Extraction and Quantitative Real-Time RCR (q-PCR)

Total RNA samples were isolated using RNAiso Plus (Takara, Shiga, Japan) and reverse transcribed to cDNA following the manufacturer’s instructions of the Primescript™ RT reagent kit with a gDNA eraser (Takara, Shiga, Japan). Diluted cDNA was used for real-time PCR with SYBR Green reagent (Vazyme, Nanjing, China) on the Roche LightCycler fluorescence quantitative PCR analyzer. The melt curves of specific primers were examined to confirm their specificities. The sequences of the primers are listed in Table S4. Expression data were normalized to the expression of β-actin. Changes in the expression were calculated using the ΔΔCt method and expressed as a fold change over the control.

### 2.9. Measurement of Oxygen Consumption and Extracellular Acidification

ECAR and OCR of MEFs experiments were conducted as described previously [23,30]. Briefly, both the ECAR and OCR of MEFs were measured using the XFe extracellular flux analyzer (Seahorse Bioscience) by plating 20,000 cells per well into an XF24 cell culture microplate with five replicates. Cell culture medium was replaced with XF-DMEM medium containing 2 mM L-glutamine, 2 mM sodium pyruvate, and 25 mM glucose. For ECAR analysis, the following reagents were applied: 10 mM glucose, 1 µM oligomycin, and 100 mM 2-DG (Seahorse Bioscience, TX, USA). And as for OCR analysis, 1 µM oligomycin, 0.5 µM FCCP, and 1 µM rotenone and antimycin (Seahorse Bioscience, TX, USA) were applied. The results were normalized using total protein determined via the PierceTM BCA Protein Assay kit (Thermo).

### 2.10. Measurement of Glucose Uptake and Lactate Production

HEK-293T cells transfected with the expression vector of PFKFB3-HK2-GLUT1 or LDHA-PDK1 were culture treated with doxycycline, and the culture medium supernatants were collected for detection. Glucose uptake analyses were measured using a glucose assay kit with O-toluidine (Beyotime, Shanghai, China), and lactate production analyses were measured using a whole-blood LD kit (Nanjing Jiancheng Bioengineering Institute, Nanjing, China). Assays were performed according to the manufacturer’s instructions. Fluorescence was measured using the Microplate Reader (BioTek Synergy H1, USA).

### 2.11. Statistical Analysis

All experiments were performed with at least three biological replicates. Experiments involving cultured cells were carried out with at least three independent replicates, and in vivo data were obtained from at least three mice for each genotype, unless otherwise stated. In some cases, representative images or results were displayed. All values were expressed as mean ± SEM. GraphPad Prism 8 software was used for the statistical analysis, and an unpaired Student’s *t*-test was used to compare the sequential measurements of the two independent groups or pairs. All *p* values less than 0.05 were considered statistically significant. In general, * means *p* < 0.05, ** means *p* < 0.01, and *** means *p* < 0.001. For animal experiments, animal numbers were kept as small as possible but still produced statistically valid data.

## 3. Results

### 3.1. Generation of BAC Transgenic Mice with the Controlled Expression of Two Sets of Glycolytic Enzymes

We previously described the design and generation of the polycistronic transgene *PFKFB3-HK2-GLUT1* [21]. The transgenic vector comprises a core expression cassette with the CMV early enhancer/chicken beta actin (CAG) promoter [31], reverse tetracycline-controlled trans-activator (rtTA) and tetracycline-responsive element (TRE) [32], a loxP-flanked transcriptional stop cassette (loxP-STOP-loxP; LSL), WPRE [33], and insulators [34]. The small 2A peptide sequences were added between genes to allow for the efficient, stoichiometric, and concordant production of discrete protein products [35]. EGFP was added at the end of polycistron. In addition, two short arms with Rosa 26 sequences flanked the expression cassette for the later recombination of the vector into the Rosa26 BAC. In this study, a transgenic vector with the polycistronic expression of *LDHA* and *PDK1* was similarly generated (Figure S1a,b).

**Figure 1:**
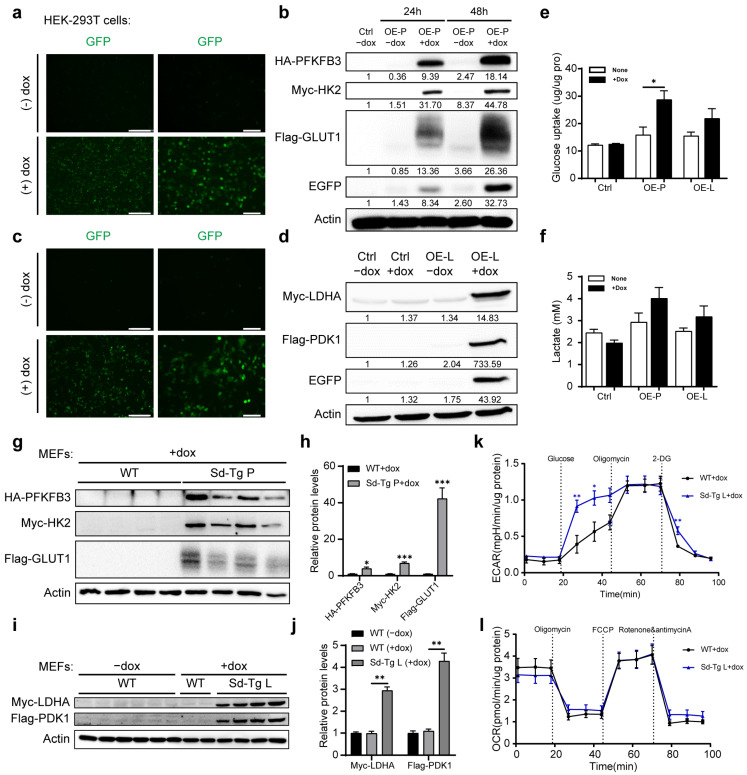
The overexpression of 2 sets of glycolytic enzymes promoted glycolysis in HEK-293T and MEF cells. (**a**) The GFP fluorescence of the OE-P HEK-293T cells with or without 1 µg/mL doxycycline treatment. Scale bar, 100 µm. (**b**) Western blot analysis of the OE-P HEK-293T cells with or without doxycycline treatment for 24 h and 48 h. (**c**) The GFP fluorescence of the OE-L HEK-293T cells with or without doxycycline treatment. Scale bar, 100 µm. (**d**) Western blot analysis of the OE-L HEK-293T cells with or without doxycycline treatment. (**e**) Glucose uptake of the OE-P or OE-L HEK-293T cells with or without doxycycline treatment. (**f**) Lactate production of the OE-P or OE-L HEK-293T cells with or without doxycycline treatment. (**g**,**h**) Western blot analysis of MEFs from Sd-Tg P mice. (**i**,**j**) Western blot analysis of MEFs from Sd-Tg L mice. (**k**) ECAR of Sd-Tg L MEFs under basal condition and followed by the sequential addition of glucose, oligomycin, and 2-DG under the treatment of doxycycline. *n* = 5. (**l**) OCR of Sd-Tg L MEFs under basal condition and followed by the sequential addition of oligomycin, FCCP, and rotenone and antimycin A under the treatment of doxycycline. *n* = 5. Values are means ± SEMs, * *p* < 0.05; ** *p* < 0.01; *** *p* < 0.001 (*t* test).

We tested the expression of the tag-fused transgenic constructs in vitro. We transfected the STOP-excised vectors into HEK-293T cells overexpressing either PFKFB3-HK2-GLUT1 (referred to as OE-P) or LDHA-PDK1 (referred to as OE-L). Microscopy for EGFP fluorescence and Western blot with the tag antibodies all exhibited the selective expression of the transgenes in the presence of doxycycline (dox) (Figure 1a–d). As the expression of the transgenes may increase the rate of glycolysis, we examined the glucose uptake and lactate production of the transfected HEK-293T cells. The OE-P cells consumed more glucose in the presence of doxycycline than the control HEK-293T cells, and both OE-P and OE-L cells appear to produce more lactate, although the effects were moderate (Figure 1e,f). Linearized transgenic vectors were used for homologous recombination into the Rosa26 BAC backbone, and pronuclear micro-injection of the mouse zygote was performed with the recombined BAC as previously described [21]. The F1 progeny from selected transgenic lines was examined using Southern blot for copy number analysis (Figure S1c,d). The expressions of the transgenes were validated via Western blot performed in mouse embryonic fibroblast cells (MEFs) derived from the STOP-deleted transgenic mice (referred to as Sd-Tg) under the treatment of doxycycline (Figure 1g–j). The Sd-Tg *PFKFB3-HK2-GLUT1* MEFs exhibited increased levels of both glucose-promoted ECAR and oligomycin-mediated maximal ECAR, whereas the OCR did not change compared with the control MEFs, as previously described [21]. The Sd-Tg *LDHA-PDK1* MEFs also showed elevated glucose-promoted ECAR levels, indicating an increase in lactate production, but with no alterations in maximal ECAR and OCR compared with the control MEFs (Figure 1k,l).

### 3.2. The Overexpression of Glycolytic Enzymes Did Not Influence Tumor Development at PanIN2 Stage

**Figure 2:**
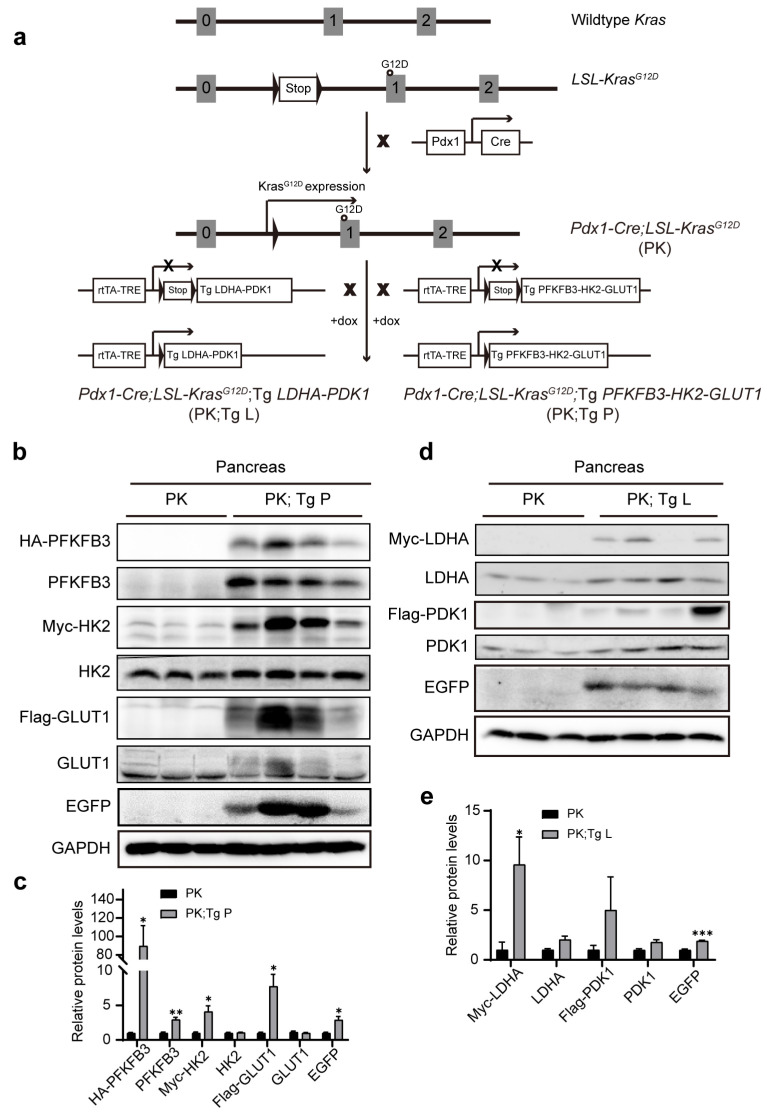
The pancreatic expression of the 2 sets of glycolytic enzymes in a mouse model of pancreas ductal tumor development. (**a**) *Pdx1-Cre mice*; *LSL-Kras^G12D^* mice (PK) and Tg P or Tg L mice were crossed to obtain PK; Tg P or PK; Tg L mice, respectively. The numbers represent exons of Kras. (**b**–**e**) Western blot analysis of pancreas tissues of PK, PK; Tg P (**b**,**c**) and PK; Tg L (**d**,**e**) mice fed drinking water containing 0.2% doxycycline starting at the age of 1 month for 3.5 months. PK mice, *n* = 3. PK; Tg P mice, *n* = 4. PK; Tg L mice, *n* = 4. * *p* < 0.05; ** *p* < 0.01; *** *p* < 0.001 (*t* test).

We next aimed to analyze the effects of the overexpression of the two sets of glycolytic enzymes on the development of PanINs and PDAC. *Pdx1-Cre; LSL-Kras^G12D^* (referred to as PK) mice were crossed with Tg *PFKFB3-HK2-GLUT1* (referred to as Tg-P) and Tg *LDHA-PDK1* (referred to as Tg-L) mice, respectively, to obtain *Pdx1-Cre; LSL-Kras^G12D^*; Tg *PFKFB3-HK2-GLUT1* (referred to as PK; Tg P) and *Pdx1-Cre; LSL-Kras^G12D^*; Tg *LDHA-PDK1* (referred to as PK; Tg L) mice. Upon the treatment of doxycycline, the expression of most of the tag-fused transgenes was elevated in the pancreas of PK; Tg mice (Figure 2 and Figure S1e,f).

**Figure 3:**
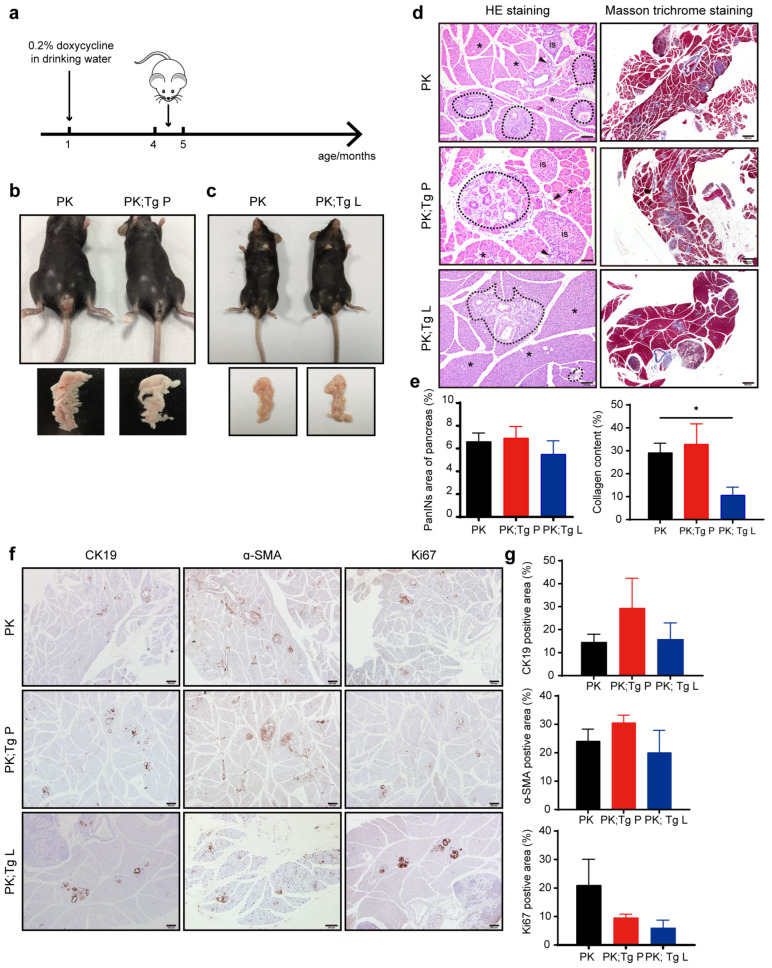
The overexpression of the glycolytic enzymes did not significantly influence tumor development at PanIN2 stage. (**a**) Mice were fed with 0.2% doxycycline in drinking water starting at the age of 1 month, and samples were collected from 4–5-month-old mice. (**b**,**c**) Photographs of the pancreases of 4–5-month-old mice. (**d**) H&E staining and Masson trichrome staining of pancreatic tissue sections revealing abnormal PanINs lesions (dashed box), ductal epithelium (arrowhead), islet (is), and surrounding acinar tissue (asterisk). *n* = 3. Scale bar, 100 µm. (**e**) Quantification of the area of PanIN lesions and collagen content of PK, PK; Tg P, and PK; Tg L mice at the age of 4–5 months in (**d**). (**f**,**g**) Immunohistochemical staining of pancreatic tissue sections for CK19, Ki67, and α-SMA. *n* = 3. Scale bar, 200 µm. Values are means ± SEMs, * *p* < 0.05 (*t* test).

Pancreatic ductal tumors slowly evolve through several distinct stages according to the differences in cell and tissue morphology in the *Pdx1-Cre; LSL-Kras^G12D^* mice, including PanIN1A/1B, PanIN2, and PanIN3 stages [4,8]. PanIN1A/1B and PanIN2 lesions occurred at 2.25 and 4.5 months of age in mice, respectively, and 9-month-old mice showed a high degree of malignant PanIN3 [9,10]. We analyzed the pathological features of the three groups of mice at ages of 4–5 months, all of which were fed drinking water containing 0.2% doxycycline starting at the age of 1 month (Figure 3a). We found no significant differences in the appearance of mice and pancreas tissue in the 4–5-month-old mice of all three groups (Figure 3b,c). Hematoxylin and eosin (H&E) staining showed clear PanIN lesions among the normal pancreatic tissues compared with the pancreases in the wild-type mice, but no differences in the PanIN lesion areas were found among the three groups (Figure S1g and Figure 3d,e). Masson trichrome staining of fibrillar collagens, an extracellular matrix component, showed a decrease in PK; Tg L mice compared to PK mice (Figure 3d,e). Cytokeratin 19 (CK19) is a protein specifically expressed by pancreatic ductal epithelial cells, and the expression level of CK19 indicates the extent of pancreatic ductal tumors [10,36]. IHC analysis demonstrated no significant differences in the CK19-positive pancreatic ductal cell population among the three mouse groups. Both α-SMA (extracellular matrix component) and Ki67 (cell proliferation marker) staining also showed no differences (Figure 3f,g). These results indicated that the overexpression of glycolytic enzymes did not further promote tumorigenesis at the early to middle stages of PanINs.

### 3.3. The Overexpression of the Two Sets of Glycolytic Enzymes Exhibited Completely Different Influences on the PanIN3 Stage of Tumor Development

**Figure 4:**
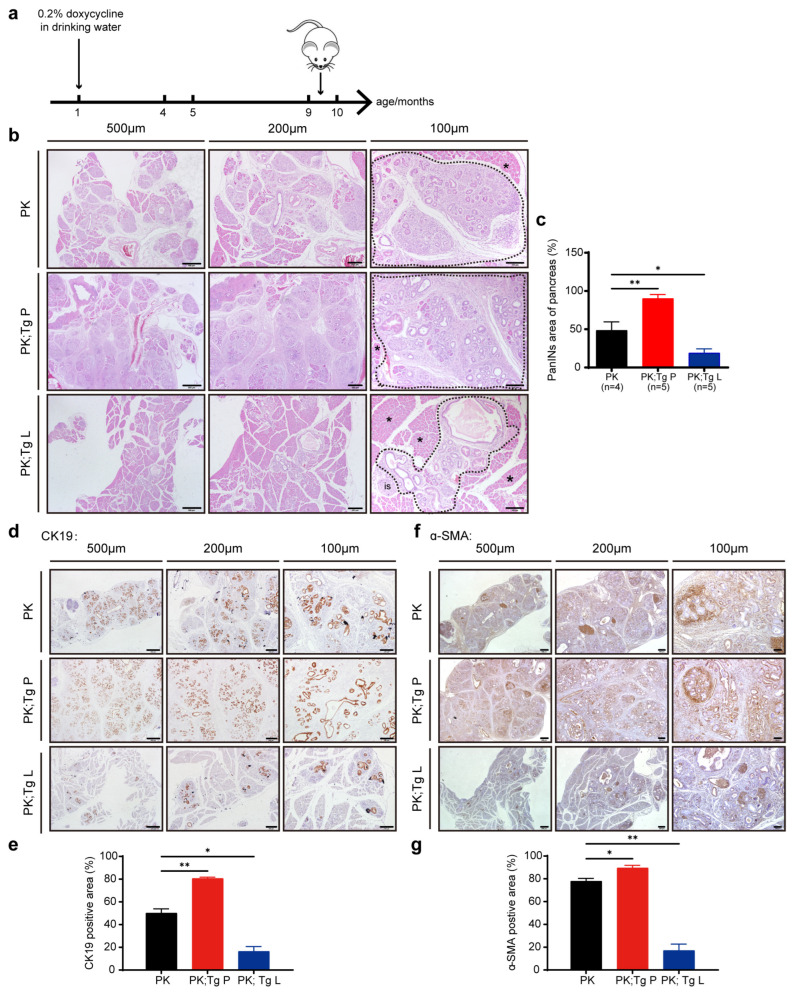
The overexpression of the 2 sets of glycolytic enzymes exhibited completely different influences at the PanIN3 stage of PDAC progression. (**a**) Mice were fed with 0.2% doxycycline in drinking water starting at the age of 1 month, and samples were collected from 9–10-month-old mice. (**b**) H&E staining of pancreas tissues from PK, PK; Tg P, and PK; Tg L mice at the age of 9–10 months revealing large PanINs lesions (dashed box), islet (is), and surrounding acinar tissue (asterisk). (**c**) Quantification of affected pancreatic areas of PK, PK; Tg P, and PK; Tg L mice at the age of 9–10 months. PK mice, *n* = 4; PK; Tg P mice, *n* = 5; PK; Tg L mice, *n* = 5. (**d**–**g**) Immunohistochemical analysis and quantification of pancreatic tissue sections from PK, PK; Tg P, and PK; Tg L mice at the age of 9–10 months for CK19 (**d**,**e**) and α-SMA (**f**,**g**). *n* = 3. For (**b**,**d**,**f**), scale bar in left, 500 µm; scale bar in middle, 200 µm; scale bar in right, 100 µm. Values are means ± SEMs, * *p* < 0.05; ** *p* < 0.01 (*t* test).

We further analyzed the pathological phenotypes of the three groups of mice at the age of 9–10 months, all of which were fed with drinking water containing 0.2% doxycycline starting at the age of 1 month (Figure 4a). Via histological analysis, we found significantly increased areas of PanIN lesions in PK; Tg P mice compared with PK mice, consistent with the sclerosis phenotype observed during tumor dissection (Figure 4b,c and Figure S2a). The pancreas/body weight ratio was also increased in PK; Tg P mice, although there were no differences in body weight among the three groups of mice (Figure S2b,c). Surprisingly, the areas of PanIN lesions were significantly reduced in PK; Tg L mice compared with PK mice (Figure 4b,c). Moreover, the IHC analyses of CK19 and Ki67 were largely consistent with the contrasting phenotypes of enhanced tumor development in the PK; Tg P mice and attenuated tumor development in PK; Tg L mice compared with PK mice (Figure 4d,e and Figure S2d,e). The Masson trichrome staining of collagen content and IHC analysis of α-SMA also exhibited extracellular matrix phenotypes corresponding to the PanIN3 development in the different strains of mice (Figure S2f,g and Figure 4f,g). These results indicated the distinct effects of the two groups of glycolytic enzymes in the late PanIN stage of tumor progression.

## 4. Discussion

Altered metabolic regulation has long been observed in human cancer, including the regulation of metabolic enzymes by frequently mutated cancer genes and the change in the expression of the enzymes and transporters of the major metabolic pathways in cancer [6,37]. The overexpressed enzymes involved in tumor glycolysis included PFK1, HK2, PDK1, LDHA, LDHB, ENO2, PKM1, and PKM2 [17,18], in addition to the glucose and lactate transporters, GLUT1, MCT1, and MCT4 [38]. Yet, the specific advantages that cancer cells acquired by undergoing metabolic reprogramming, and the key molecular determinants of the metabolic reprogramming, are likely highly complex. For example, the contributions of various glycolytic enzymes or distinct metabolic routes in promoting metabolic programming and tumorigenesis are not well understood.

In this study, we utilized two strains of BAC transgenic mice, Tg *PFKFB3-HK2-GLUT1* and Tg *LDHA-PDK1*, which express two distinct sets of critical glycolytic enzymes found in human cancers, to address the possible effects of differential glycolytic programming in promoting pancreatic ductal tumor development. We found that the maximal glycolysis capacity of cells overexpressing PFKFB3-HK2-GLUT1 was greatly enhanced, whereas the maximal glycolysis capacity of cells overexpressing LDHA-PDK1 remained unchanged, although the glucose-stimulated lactate production was increased in both cases. These results were consistent with an enhanced glucose uptake and overall increased glycolytic flux in the former and only an altered pyruvate fate away from mitochondria in the latter. Indeed, the manipulation of LDHA and PDK1 in nerve cells was shown to decrease mitochondrial oxidative metabolism and ROS levels [25]. Thus, the differential regulation of glycolysis by the two sets of transgenes may provide novel opportunities to explore the critical determinants in glycolytic metabolic programming in promoting tumorigenesis.

By analyzing the effects of the transgenic expression of the two sets of glycolytic enzymes on the development of PanINs in mice, we found the PFKFB3-HK2-GLUT1 transgene dramatically accelerated the growth of PanIN3 lesions, corroborating with previous studies to indicate a direct role of enhanced glycolysis in promoting tumorigenesis. The promoting effect was only limited at the PanIN3 stage, suggesting that enhanced metabolic programming might be more critical when tumor growth is accelerated or at the transition to a more malignant state.

Several lines of evidence support the conclusion that LDHA and PDK1 are important potential therapeutic targets in different cancer types, and that their inhibition can prove beneficial to reduce the growth of several cancer cell types, including PDAC [39,40,41]. LDHA expression is important to regenerate NAD^+^ to sustain the glycolytic flux, while PDK1 may further promote glycolysis by limiting pyruvate entry into mitochondria [42,43]. Surprisingly, the transgenic expression of LDHA-PDK1 suppressed the progression of PanIN at stage 3. It is plausible that while this transgene did not improve the glucose influx into the tumor cells, it might have severely disturbed the metabolic flux into TCA cycle, resulting in a metabolic defect that impaired the fast growth of the tumor. TCA metabolites and their derivatives are critical for fatty acid and nucleotide syntheses, which are crucial for cell proliferation [44]. The coordinated elevation of the multiple enzymes in the glycolytic pathways found in tumors may be the key to promoting glycolysis while allowing for the limited but significant entry of pyruvate into mitochondria from the highly increased metabolic flux. Further analysis on the metabolic flux in these tumors is warranted to address the question. Nonetheless, our results strongly suggested that enhanced glycolytic flux/capacity and a balance between glycolysis and mitochondrial metabolism may both be critical in glycolytic reprogramming for the promotion of tumorigenesis.

From therapeutic perspectives, as our results showed that the responses to increased glycolysis at the early and late stages of pancreatic ductal tumorigenesis were different, it would be interesting to examine whether targeting glycolysis would affect tumor development differentially at various stages, and thus gain insight into targeting metabolism with precision. Moreover, our studies implicated that the metabolic flux into mitochondria may be critical during metabolic reprogramming in the promotion of tumorigenesis. Therefore, highly glycolytic tumor cells might exhibit a vulnerability in mitochondrial metabolism that can be explored for targeting. Further, while the inhibition of LDHA or PDK1 reduces glycolysis, it might instead elevate mitochondria-related biosynthetic pathways for tumor cell proliferation or survival. Taking into consideration mitochondria-related pathways when targeting glycolysis might help to design strategies for better therapeutic effects.

## 5. Conclusions

**Figure 5:**
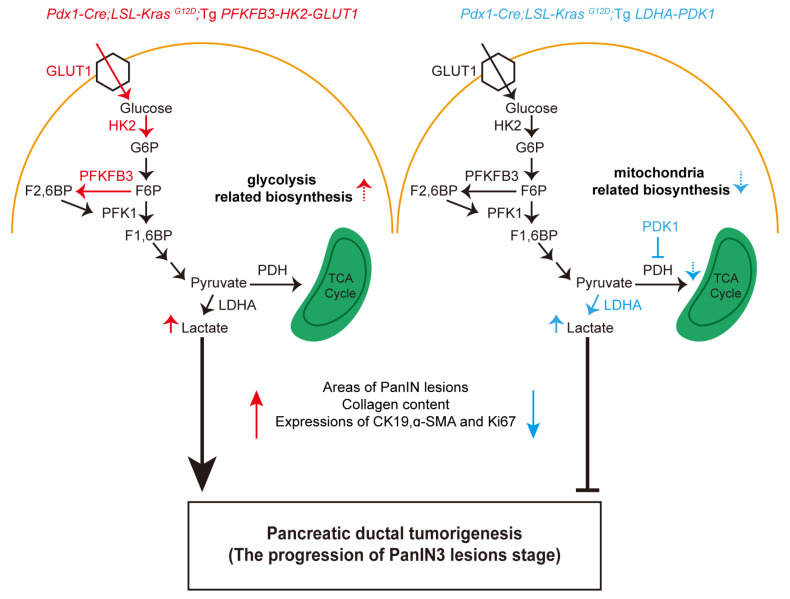
The expression of two distinct sets of glycolytic enzymes that are involved in different glycolytic routes exhibited contrasting effects on pancreatic ductal tumorigenesis in mice. G6P, Glucose-6-phosphate; F6P, Fructose-6-phosphate; F1,6P, Fructose-1,6-bisphosphate; F2,6P, Fructose-2,6-bisphosphate.

The present study finds that the overexpression of glycolytic enzymes PFKFB3-HK2-GLUT1 significantly accelerated the progression of the PanIN3 lesions stage in 9–10-month-old *Pdx1-Cre; LSL-Kras^G12D^* mice, exhibiting elevated levels of ductal cell marker CK19 and matrix components. Surprisingly, the overexpression of LDHA-PDK1 significantly attenuated the progression of the PanIN3 lesions stage in 9–10-month-old mice with significantly downregulated levels of CK19, cell proliferation marker Ki67, and the matrix components (Figure 5). Thus, the two distinct sets of glycolytic enzymes that are involved in different glycolytic routes exhibited contrasting effects on pancreatic ductal tumorigenesis in mice. These results strongly suggested that enhanced glycolytic flux/capacity and a balance between glycolysis and mitochondrial metabolism may both be critical in glycolytic reprogramming in the promotion of tumorigenesis. It is important to note that a limitation of the present study is the absence of further research on metabolic flux/state and signaling pathways. Nonetheless, our manipulation and identification of the differential glycolytic routes and their influences on pancreatic tumor development should provide new insights into cancer metabolic reprogramming and its potential targeting for cancer therapy.

## Data Availability

Data presented in this manuscript are available upon request from the corresponding authors.

## Supplementary Materials

The following supporting information can be downloaded at: https://www.mdpi.com/article/10.3390/biomedicines11112962/s1, Table S1: Primer information for genotyping; Table S2: The list of antibodies used for IHC in this study; Table S3: The list of antibodies used for WB in this study; Table S4. Primer information for q-PCR analysis of expression of target genes. Figure S1: Construction of transgenic vectors and generation of the BAC transgenic mice; Figure S2: The overexpression of the 2 sets of glycolytic enzymes differentially influenced pancreatic ductal tumor progression. Reference [45] is cited in Supplementary Materials.
