# Supplementary material for: The Expression of Two Distinct Sets of Glycolytic Enzymes Reveals Differential Effects of Glycolytic Reprogramming on Pancreatic Ductal Tumorigenesis in Mice
Source: Biomedicines. 2023 Nov 2;11(11):2962. doi: 10.3390/biomedicines11112962 (PMC10669313; doi:10.3390/biomedicines11112962)
Supplement: Supplementary file 1 [file biomedicines-11-02962-s001.zip › biomedicines-2619647-supplementary.pdf]

biomedicines MDPI

The expression of two distinct sets of glycolytic enzymes reveals differential effects of glycolytic reprogramming on pancreatic ductal tumorigenesis in mice

Supplementary Materials:

Table S1. Primer information for genotyping.

Genes	Forward primers 5'-->3'	Reverse primers 5'-->3'
EIIA-Cre	AATGCTTCTGTCCGTTTGC	ACCAGAGTCATCCTTAGCG
Pdx1-Cre	AATGCTTCTGTCCGTTTGC	ACCAGAGTCATCCTTAGCG
$Kras^{G12D}$	AGCTAGCCACCATGGCTTGAG-TAAGTCTGCA	CCTTTACAAGCGCACGCAGACTG-TAGA
BAC-Tg P	GTCGACTGCAGTTAATTAAC	TGCGTCAGTTCCAACGGCATG
BAC-Tg L	GTCGACTGCAGTTAATTAAC	GCTGATCCTTTAGAGTTGCC

Table S1. The list of antibodies used for IHC in this study.

Antibody name	Company	Cat No.	Source
anti-CK19	Abcam	ab52625	Rabbit
anti-Ki67	Santa Cruz	sc-7864	Goat
anti-α-SMA	Abcam	ab7817	Mouse
anti-CK19	Abcam	ab52625	Rabbit

Table S3. The list of antibodies used for WB in this study.

Antibody name	Company	Cat No.	Source
anti-HA	Sigma	H9658	Mouse
anti-PFKFB3	Abcam	ab181861	Rabbit
anti-Myc	Santa Cruz	sc-40	Mouse
anti-HK2	Santa Cruz	sc-6521	Goat
anti-Flag	Sigma	F3165	Mouse
anti-GLUT1	Abcam	ab652	Rabbit
anti-LDHA	Santa Cruz	sc-27230	Goat
anti-PDK1	Santa Cruz	sc-7141	Goat
anti-Actin	Abclonal	AC026	Rabbit
anti-GFP	Santa Cruz	Sc-8334	Rabbit
anti-α-Tubulin	Abclonal	AC012	Mouse
anti-GAPDH	Abclonal	AC001	Rabbit
HRP Goat anti-mouse IgG	Abclonal	AS003	Goat
HRP Goat anti-Rabbit IgG	Abclonal	AS014	Goat
HRP Rabbit anti- Goat IgG	Abclonal	AS029	Rabbit

Table S4. Primer information for q-PCR analysis of expression of target genes.

Genes	Forward primers 5'-->3'	Reverse primers 5'-->3'
β-actin	CCTGTATGCCTCTGGTCGTA	CCATCTCCTGCTCGAAGTCT
HA-PFKFB3	GCTCTAGAGATATCAC CATGGGGCCCGGCTAT	AAGGTCAAAATTCAAAGTCTGTTTCAC- TCCGCTTCCGTGTTTCCTGGAGGAGTCA
Myc-HK2	GGAATTCATGATTGC CTCGCATCTGCTTGC	CCTGCTTCAGCAGGCTGAAGTTAG- TAGCTCCGCTTCCTCGCTGTCCAGCCTCACG
Flag-GLUT1	ATAAGAATGCGGCCGCCAT GGAGCCCAGCAGCAAGAA	AGCAGACTTCCTCTGCCC TCTCCGCTTCCCACTTGGGAATCAGCCCC
Myc-LDHA	GGAATTCATGGCAACTCTAAAGGATCAG	CTCCAGCCAATTTCAAGAGAGCATAATTA GTACACTGAAATTGCAGCTCCTTTTGGATC
Flag-PDK1	ATAAGAATGCGGCCGCCATG AGGCTGGCGCGGCTGCTT	AGCAGACTTCCTCTGCCCTCTCCGCTTCC GGCACTGCGGAACGTCGTCATG

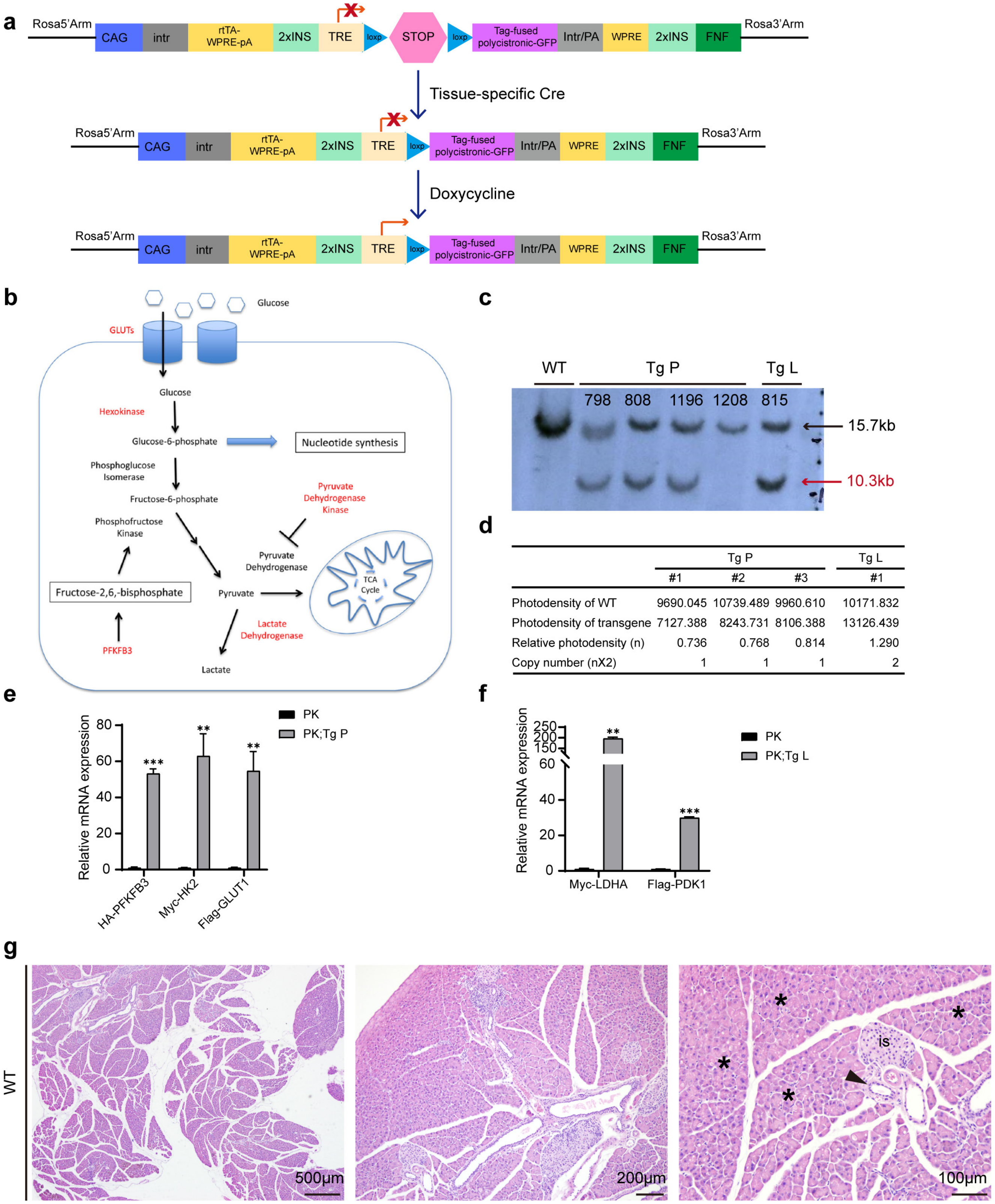

	Tg P			Tg L
	#1	#2	#3	#1
Photodensity of WT	9690.045	10739.489	9960.610	10171.832
Photodensity of transgene	7127.388	8243.731	8106.388	13126.439
Relative photodensity (n)	0.736	0.768	0.814	1.290
Copy number (nX2)	1	1	1	2

Figure S1. Construction of transgenic vectors and generation of the BAC transgenic mice. (a) Schematic representations of the strategy for controlled expression of tag-fused polycistronic genes in Rosa26 BAC transgenic mice. (b) The diagram showing glycolytic pathways and related metabolic enzymes [45]. The glycolytic enzymes overexpressed in the transgenic mice were marked in red. (c)

Southern blot analysis of the selected founder lines of the transgenic mice. By MscI-digestion and 3′ probe hybridization, WT group displayed one band corresponding to 15.7 kb, while Tg P group and Tg L group displayed two bands corresponding to 15.7 kb and 10.3 kb. Numbers indicated transgenic lines. (d) Quantification of the band density in (c) to estimate the BAC transgene copy number. (e) q-PCR analysis of expression of tag-fused transgenes in pancreas tissues of PK, PK; Tg P mice feeding with drinking water containing 0.2% doxycycline starting at the age of 1 month for 3.5 months. PK mice, n=4. PK; Tg P mice, n=3. (f) q-PCR analysis of expression of tag-fused transgenes in pancreas tissues of PK, PK; Tg L mice feeding with drinking water containing 0.2% doxycycline starting at the age of 1 month for 3.5 months. PK mice, n=2. PK; Tg L mice, n=2. (g) Different magnifications of H&E stained pancreas tissue in wild-type mouse showing normal ductal epithelium in cross-section (arrowhead), islet (is), and surrounding acinar tissue (asterisk). Values are means ± SEMs, **p*< 0.05; ***p* < 0.01; ****p* < 0.001 (t test).

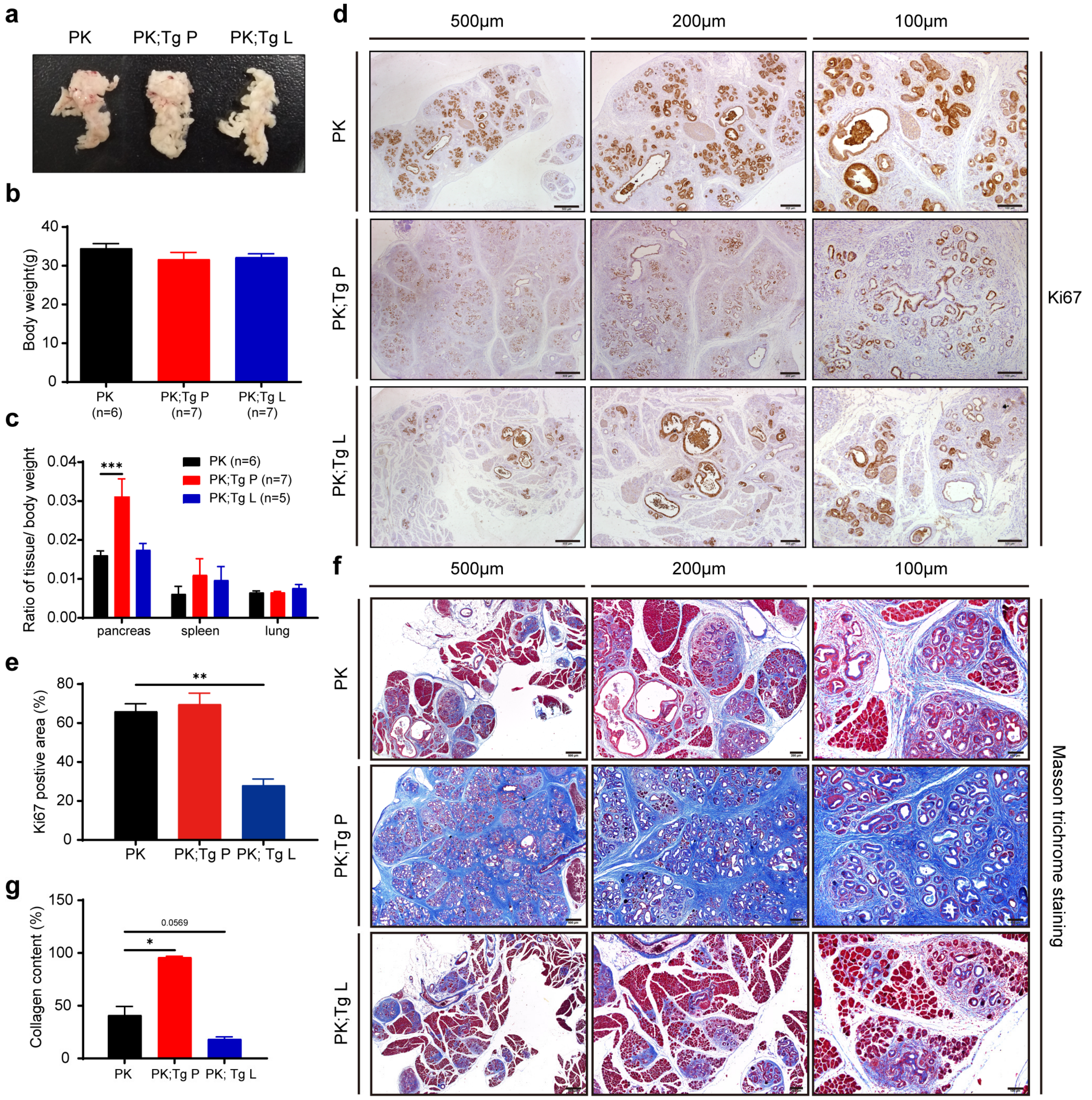

Figure S2. The overexpression of the 2 sets of glycolytic enzymes differentially influenced pancreatic ductal tumor progression. (a) Photographs of the pancreas of PK, PK; Tg P and PK; Tg L mice

at the age of 9-10 months. (b) Body weights of PK mice (n=3), PK; Tg P mice (n=7) and PK; Tg L mice (n=3) at the age of 9-10 months. (c) Ratio of tissues/body weight of PK mice (n=6), PK; Tg P mice (n=7) and PK; Tg L mice (n=5) at the age of 9-10 months. (d, e) Immunohistochemical analysis and quantification of pancreatic tissue sections from PK, PK; Tg P and PK; Tg L mice at the age of 9-10 months for Ki67. n=3. Scale bar in left, 500 μm; scale bar in middle, 200 μm; scale bar in right, 100 μm. (f, g) Masson's trichrome staining and quantification of pancreatic tissue sections from PK, PK; Tg P and PK; Tg L mice at the age of 9-10 months. n=3. Scale bar in left, 500 μm; scale bar in middle, 200 μm; scale bar in right, 100 μm. Values are means ± SEMs, *$p < 0.05$; **$p < 0.01$; ***$p < 0.001$ (t test).
